# Supplementary material for: Directed natural evolution generates a next-generation oncolytic virus with a high potency and safety profile
Source: Nat Commun. 2023 Jun 9;14:3410. doi: 10.1038/s41467-023-39156-3 (PMC10256765; doi:10.1038/s41467-023-39156-3)
Supplement: Supplementary file 1 — Supplementary Information [file 41467_2023_39156_MOESM1_ESM.pdf]

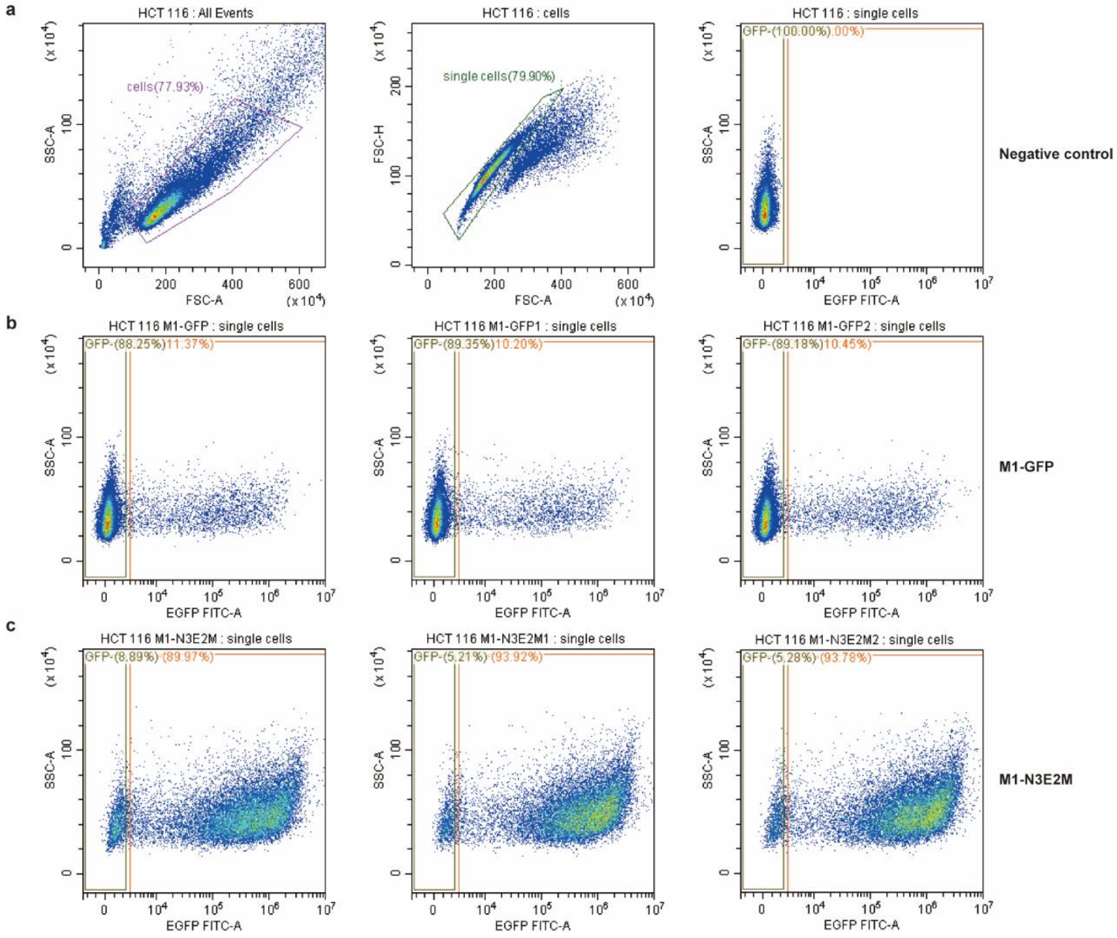

Fig. S1. Infection rate of M1 viruses on HCT-116 cell line. HCT-116 cells were infected with M1-GFP and M1-N3E2M (MOI=1) for 48 hours, and the infection rate was determined by flow cytometry. **a** The gating strategy. **b** Flow cytometry analysis showing the levels of GFP^{+} cells after infection with M1-GFP. n = 3 biological replicates. **c** Flow cytometry analysis showing the levels of GFP^{+} cells after infection with M1-N3E2M. n = 3 biological replicates.

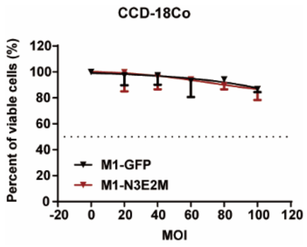

Fig. S2. Killing effects of M1 viruses on normal cell lines. CCD-18Co cells were treated with M1-GFP and M1-N3E2M (MOI=20, 40, 60, 80and 100). EC50 shift was calculated by nonlinear regression. Data points represent mean % viability relative to vehicle ± SD, for n = 3 biological replicates. Source data are provided as a Source Data

file.

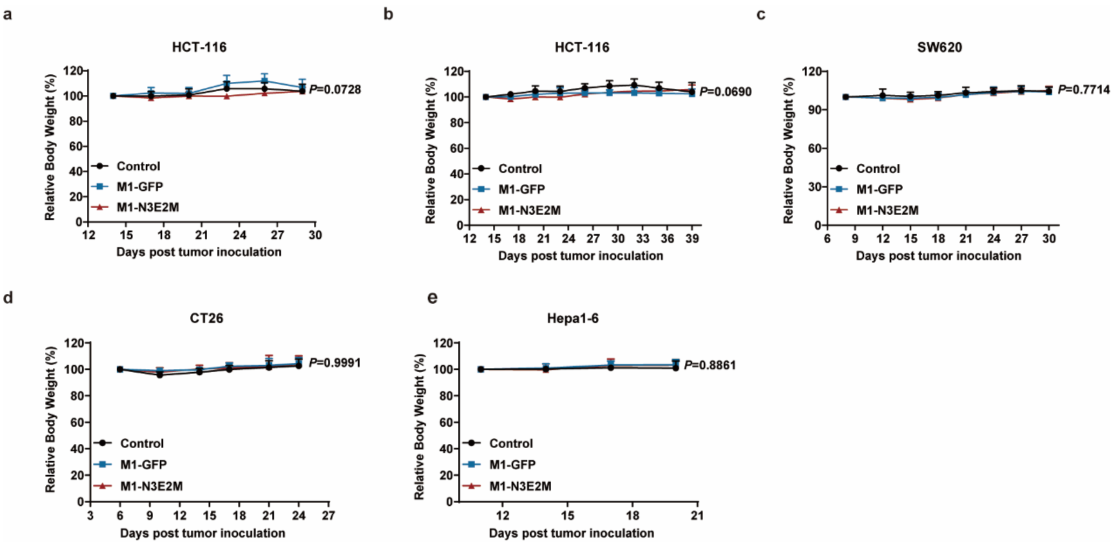

Fig. S3. The body weights of mice in vivo. a Relative body weights of mice in Fig. 3a were measured every three days. n = 7 mice per group. **b** Relative body weights of mice in Fig. 3e were measured every three days. n=7 mice per group. **c** Relative body weights of mice in Fig. 3g were measured every three days. n=10 mice per group. **d** Relative body weights of mice in Fig. 3i were measured. n=5 mice per group. **e** Relative body weights of mice in Fig. 3k were measured every three days. Control n=7 mice, M1-GFP n=9 mice, M1-N3E2M n=8 mice. Data points **(a-e)** represent mean body weight relative to the body weight before treatment ± SD. Statistical significance was calculated using Two-way ANOVA and *P* values are indicated. n.s.: no significance ($P > 0.05$). Source data are provided as a Source Data file.

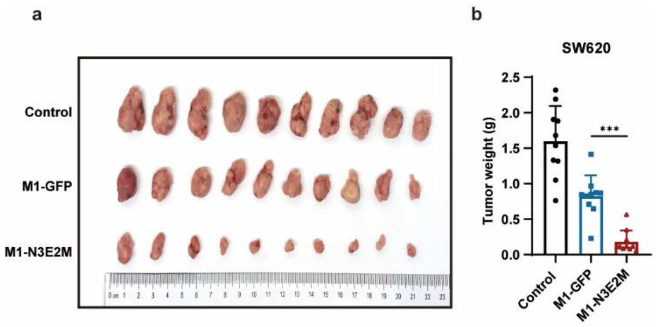

Fig. S4. Tumor weights of SW620 xenograft models. a Images of tumors from every group in Fig. 3h at the experimental endpoints. **b** Bar graph of tumor weights in every group at the experimental endpoints. Graph bars represent mean tumor weights ± SD,

for n = 10 biologically independent samples. *P* = 0.0008 was calculated using One-way ANOVA with Sidak's multiple comparisons test relative to M1-GFP. ***$P < 0.001$. Source data are provided as a Source Data file.

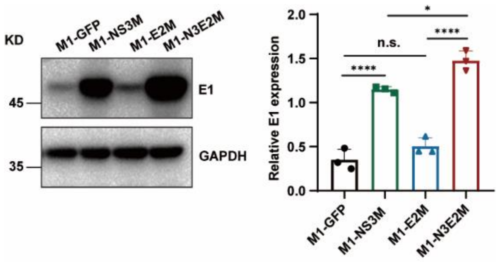

Fig. S5. The replication of M1 viruses in HCT-116 cells. The structural protein E1 was detected by Western blotting in HCT-116 cells infected with M1-GFP, M1-NS3M, M1-E2M, or M1-N3E2M at an MOI of 1 at 48 hpi (left). Quantification of E1 expression is shown (right). Graph bars represent mean densitometry of E1 ± SD, for n = 3 biological replicates. Statistical significance was calculated using One-way ANOVA with Tukey's multiple comparisons test. Adjusted *P* values are: M1-NS3M vs. M1-GFP, $P < 0.0001$; M1-E2M vs. M1-GFP, $P = 0.2913$; M1-N3E2M vs. M1-NS3M, $P = 0.0145$; M1-N3E2M vs. M1-E2M, $P < 0.0001$. n.s.: no significance *$P < 0.05$, ****$P < 0.0001$. Source data are provided as a Source Data file.

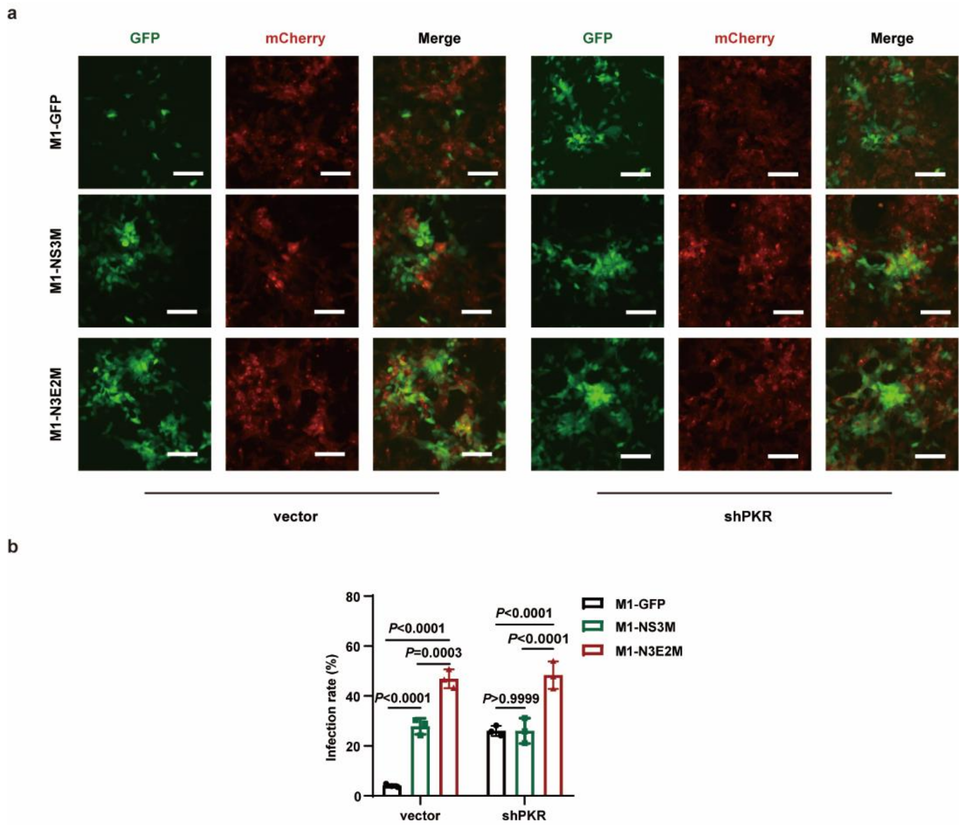

Fig. S6. The infection of M1 viruses in PKR knockdown HCT-116 cells. a PKR knockdown cells were infected with M1-GFP, M1-NS3M and M1-N3E2M at an MOI of 1, and imaged with a fluorescence microscope 24 h after infection. Representative images of n = 3. Scale bars, 50 μm. **b** The infection rate was determined by flow cytometry. Graph bars represent mean infection rate % ± SD, for n = 3 biological replicates. Statistical significance was calculated using Two-way ANOVA with Sidak's multiple comparisons test relative to M1-GFP and adjusted *P* values are indicated. Source data are provided as a Source Data file.

Supplementary tables:

Table S1. Host proteins that specifically interact with nsP3

Accession	Description
A0A0J9YW13	RNA-binding protein 8A (Fragment) GN=RBM8A
B2R858	cDNA, FLJ93750, Homo sapiens DEAD (Asp-Glu-Ala-Asp) box polypeptide 6 (DDX6), mRNA
B4DGW8	cDNA FLJ60505, highly similar to Apolipoprotein B mRNA-editing enzyme catalytic polypeptide-like 3F
B4DLY2	cDNA FLJ56600, highly similar to Large proline-rich protein BAT2
B4DMQ9	cDNA FLJ58590, highly similar to YLP motif-containing protein 1
B4E3F7	cDNA FLJ57455, moderately similar to Homo sapiens muscleblind-like 2 (Drosophila) (MBNL2), transcript variant 3, mRNA
B5MCT8	40S ribosomal protein S9 GN=RPS9
B7Z6Y2	cDNA FLJ54942, highly similar to Homo sapiens bridging integrator 1 (BIN1), transcript variant 10, mRNA
B7Z972	Protein-L-isoaspartate O-methyltransferase
C9JC24	WD repeat-containing protein 48 (Fragment) GN=WDR48
D3DWL9	Cleavage and polyadenylation specific factor 1, 160kDa, isoform CRA_a GN=CPSF1
E7EWK3	ATP-dependent RNA helicase DHX36 (Fragment) GN=DHX36
E7EX53	Ribosomal protein L15 (Fragment) GN=RPL15
E9PDY7	Pleckstrin homology-like domain family B member 2 GN=PHLDB2

E9PKG1	Protein arginine N-methyltransferase 1 GN=PRMT1
F8WE71	Serine/threonine-protein phosphatase PP1-beta catalytic subunit GN=PPP1CB
G3V1A1	60S ribosomal protein L8 GN=RPL8
G3V279	Enhancer of rudimentary homolog GN=ERH
G5EA18	Double-stranded RNA-binding protein Staufen homolog 2 GN=STAU2
H0YAF8	Receptor of-activated protein C kinase 1 (Fragment) GN=RACK1
H0YAM1	G-rich sequence factor 1 (Fragment) GN=GRSF1
H0YAP0	Zinc finger C2HC domain-containing protein 1A (Fragment) GN=ZC2HC1A
H0YIB4	Serine/arginine-rich-splicing factor 9 (Fragment) GN=SRSF9
H0YLA2	Signal recognition particle 14 kDa protein GN=SRP14
H0YLP6	60S ribosomal protein L28 GN=RPL28
H7C3M2	60S ribosomal protein L3 (Fragment) GN=RPL3
H7C4B0	DNA topoisomerase (Fragment) GN=TOP3B
I0B0K3	Truncated profilaggrin GN=FLG
J3KSY7	Protein CASC3 (Fragment) GN=CASC3
J3KT73	60S ribosomal protein L38 GN=RPL38
K7EJB5	Small nuclear ribonucleoprotein Sm D2 GN=SNRPD2
K7EJT5	60S ribosomal protein L22 (Fragment) GN=RPL22
M0QZ60	Signal-transducing adaptor protein 2 GN=STAP2
M0R0P1	rRNA 2'-O-methyltransferase fibrillarin (Fragment) GN=FBL

M4VP52	Apolipoprotein B editing enzyme catalytic polypeptide-like 3C GN=APOBEC3C
O43920	NADH dehydrogenase [ubiquinone] iron-sulfur protein 5 GN=NDUFS5
O75569	Interferon-inducible double-stranded RNA-dependent protein kinase activator A GN=PRKRA
O95782	AP-2 complex subunit alpha-1 GN=AP2A1
P04040	Catalase GN=CAT
P19474	E3 ubiquitin-protein ligase TRIM21 GN=TRIM21
P29508	Serpin B3 GN=SERPINB3
P47929	Galectin-7 GN=LGALS7
P62318	Small nuclear ribonucleoprotein Sm D3 GN=SNRPD3
Q09161	Nuclear cap-binding protein subunit 1 GN=NCBP1
Q13643	Four and a half LIM domains protein 3 GN=FHL3
Q2I377	Small proline rich protein
Q2VIL4	RcDNAJ9 (Fragment)
Q2VPJ6	HSP90AA1 protein (Fragment) GN=HSP90AA1
Q53T09	Uncharacterized protein XRCC5 (Fragment) GN=XRCC5
Q59FA2	Splicing factor, arginine/serine-rich 1 (Splicing factor 2, alternate splicing factor) variant (Fragment)
Q6NTA2	HNRNPL protein (Fragment) GN=HNRNPL
Q92615	La-related protein 4B GN=LARP4B
Q96DI9	POLDIP3 protein (Fragment) GN=POLDIP3

Q96I24	Far upstream element-binding protein 3 GN=FUBP3
Q9BRL5	CALM3 protein
Q9BYK8	Helicase with zinc finger domain 2 GN=HELZ2
Q9NZT1	Calmodulin-like protein 5 GN=CALML5
Q9Y295	Developmentally-regulated GTP-binding protein 1 GN=DRG1
S4R456	40S ribosomal protein S15 (Fragment) GN=RPS15

Accession: Protein numbering in the FASTA database. Description: Protein functional descriptions in protein sequence-based databases. GN= Gene Name.

Table S2. Host proteins that specifically interact with nsP3 M358L

Accession	Description
A0A1W2PRE1	Guanine nucleotide-binding protein G(o) subunit alpha GN=GNAO1
A1A5C5	RRBP1 protein GN=RRBP1
B2R4M6	Protein S100
B3KM36	cDNA FLJ10153 fis, clone HEMBA1003417, highly similar to BAG family molecular chaperone regulator 2
B3KME0	cDNA FLJ10760 fis, clone NT2RP3004618, highly similar to Eukaryotic translation initiation factor 2C 1
B4E1G5	cDNA FLJ53692, moderately similar to Granulins
B4E2A4	cDNA FLJ53275, highly similar to Homo sapiens spectrin domain with coiled-coils 1 (SPECC1), transcript variant, mRNA
C9JYQ9	60S ribosomal protein L22-like 1 GN=RPL22L1

E7ETK0	40S ribosomal protein S24 GN=RPS24
F4MHG5	Ubiquitously transcribed tetratricopeptide repeat protein Y-linked transcript variant 184 GN=UTY
F8W1I4	La-related protein 4 (Fragment) GN=LARP4
G3V1T9	RNA binding motif protein 7, isoform CRA_a GN=RBM7
J3QSB5	60S ribosomal protein L36 GN=RPL36
P01859	Immunoglobulin heavy constant gamma 2 GN=IGHG2
P07305	Histone H1.0 GN=H1F0
P16403	Histone H1.2 GN=HIST1H1C
P18124	60S ribosomal protein L7 GN=RPL7
P19525	Interferon-induced, double-stranded RNA-activated protein kinase GN=EIF2AK2
P62753	40S ribosomal protein S6 GN=RPS6
P98179	RNA-binding protein 3 GN=RBM3
Q8IWR8	Ribosomal protein L19 (Fragment)
Q9UG73	Uncharacterized protein DKFZp586B1222 (Fragment) GN=DKFZp586B1222

Accession: Protein numbering in the FASTA database. Description: Protein functional descriptions in protein sequence-based databases. GN= Gene Name.

Table S3. Clinical symptoms

Animal No.	Soft stool	Diarrhea	Sparse coat	wound	Scab
Excipient					

Male-1	+	+	-	-	-
Male-2	-	-	-	-	-
Female-1	-	-	-	-	-
Female-2	-	-	-	-	-
M1-N3E2M					
Male-3	-	-	-	-	-
Male-4	-	-	-	-	-
Female-3	-	-	-	-	-
Female-4	-	+	-	-	-

-= No abnormalities, += Present.

Table S4. Ophthalmological examination

Animal No.	external ocular	anterior chamber	posterior chamber	fundus
Excipient				
Male-1	-	-	-	-
Male-2	-	-	-	-
Female-1	-	-	-	-
Female-2	-	-	-	-
M1-N3E2M				
Male-3	-	-	-	-
Male-4	-	-	-	-
Female-3	-	-	-	-

Female-4	-	-	-	-

-= No abnormalities.

Table S5. Urinalysis

Animal No.	Days	clarity	pH	GLU	PRO	KET	BLD	LEU
Excipient								
Male-1	-5	Lt.Turbid	9	neg	neg	neg	5+	2+
	68	Clear	9	neg	neg	neg	neg	neg
Male-2	-5	Turbid	8	neg	2+	1+	5+	1+
	68	Lt.Turbid	8	neg	1+	neg	neg	neg
Female-1	-5	Turbid	9	neg	2+	neg	5+	3+
	68	Turbid	8	neg	2+	1+	2+	3+
Female-2	-5	Clear	8	neg	neg	neg	3+	2+
	68	Turbid	8	neg	1+	Neg	4+	3+
M1-N3E2M								
Male-3	-5	Lt.Turbid	9	neg	1+	neg	neg	neg
	68	Lt.Turbid	9	neg	1+	neg	1+	1+
Male-4	-5	Lt.Turbid	8	neg	2+	1+	3+	1+
	68	Clear	9	neg	neg	neg	4+	neg
Female-3	-5	Lt.Turbid	9	neg	1+	neg	2+	1+
	68	Lt.Turbid	9	neg	1+	neg	neg	3+
Female-4	-5	Turbid	9	neg	1+	neg	5+	3+

	68	Lt.Turbid	8	neg	neg	neg	neg	3+

GLU (Glucose) :1+ ≈ ~3 mmol/L, 2+ ≈ ~6 mmol/L, 3+ ≈ ~17mmol/L, 4+ ≈ ~56 mmol/L

KET (Ketone) :1+ ≈ ~0.5 mmol/L, 2+ ≈ ~1.5 mmol/L, 3+ ≈ ~5 mmol/L,4+ ≈ ~15 mmol/L

BLD (Blood) :1+ ≈ ~10 /μL, 2+ ≈ ~25 /μL, 3+ ≈ ~50 /μL, 4+ ≈ ~150 /μL 5+ ≈ ~250 /μL

PRO (Protein):1+ ≈ ~0.25 g/L, 2+ ≈ ~0.75 g/L, 3+ ≈ ~1.5 g/L, 4+ ≈ ~5 g/L

LEU (Leucocyte): 1+ ≈ ~25 /μL, 2+ ≈ ~100 /μL, 3+ ≈ ~500 /μL

Lt.Turbid = Light Turbid, neg=negative

Table S6. Anti-M1 antibodies in macaque serum

Animal No.	D-3	D20	D43	D72
Excipient				
Male-1	-	-	-	-
Male-2	-	-	-	-
Female-1	-	-	-	-
Female-2	-	-	-	-
M1-N3E2M				
Male-3	-	-	-	-
Male-4	-	+	-	-
Female-3	-	-	-	-
Female-4	-	+	-	-

-= Negative, +=Positive.

Table S7. Primers used for genome sequencing

	Forward Primers（F）	Reverse Primers（R）
1-1144	ATGGCGGACGTGTGACATC	TCTGGGCATCCTCTGGTG
981-2413	TTACGCAGTTACCCATCAC	CTTTCTTGCACCCATTCA
2335-3830	AATGATGTAAAGAGGCAACG	GGTCTAAGCAGGTGTAGTGAA
3544-4975	AGGGTAGAGTGGTTGCTGA	GCACGCCTTCTATCCTGT
4931-6315	TGTGCTCCTCCTTTCCAT	AAACACCGCAGAGTCCA
6084-7524	CGCTTATCTGGACTTGGT	TGTCTGTAATACGTGGTCTTTA
7375-PR1	AGTGGCGTCGGGAACATC	AACAGCTCCTCGCCCTTGCTCACCA
PF2-8809	ATGGTGAGCAAGGGCGAGGAGCTG T	ATCGTGCCTCGAATAGCG
8642-10136	TTCCGATGGCATGATAAA	CGGCACGACTGTCTTGTA
9936-11696	TTGGTGAGCCTGGGAAC	GTAAAATATTAAAAAAACAAATTA GACG

The primers were synthesized at Thermo Fisher Scientific company.

Table S8. Cell line

Name	Cat no.	source	STR identification	Mycoplasma contamination	application
Sk-HEP-1	HTB-52	ATCC	Correct	Negative	Fig. 2a, b
HCT-116	CCL-247	ATCC	Correct	Negative	Fig. 1, 2a-b, 3a-f, 4, 5a-c,

					6a-o, S1, S3a-b, S5, S6,
ZR-75-1	CRL-1500	ATCC	Correct	Negative	Fig. 2a, b
HCT-8	100308	BNCC	Correct	Negative	Fig. 2a, c, d
RT112/84	RT112/84	NTCC	Correct	Negative	Fig. 2a, b
MCF-7	HTB-22	ATCC	Correct	Negative	Fig. 2a, b
Capan-2	HTB-80	ATCC	Correct	Negative	Fig. 2a, b
Huh 7	337690	BNCC	Correct	Negative	Fig. 2a, b, e, f
BT-20	HTB-19	ATCC	Correct	Negative	Fig. 2a, b
MDA-MB-435S	HTB-129	ATCC	Correct	Negative	Fig. 2a, b
HPAC	CRL-2119	ATCC	Correct	Negative	Fig. 2a, b
PANC-1	CRL-1469	ATCC	Correct	Negative	Fig. 2a, b
HT-29	HTB-38	ATCC	Correct	Negative	Fig. 2a, b
MIA PaCa-2	CRL-1420	ATCC	Correct	Negative	Fig. 2a, b
HCC38	CRL-2314	ATCC	Correct	Negative	Fig. 2a, b
HT-1376	CRL-1472	ATCC	Correct	Negative	Fig. 2a, b
SW620	CCL-227	ATCC	Correct	Negative	Fig. 2a-b, 3g-h, S3c, S4,
SW 1990	CRL-2172	ATCC	Correct	Negative	Fig. 2a, b
T-47D	HTB-133	ATCC	Correct	Negative	Fig. 2a, b
DU 145	HTB-81	ATCC	Correct	Negative	Fig. 2a, b
VM-CUB-1	CVCL_1786	NTCC	Correct	Negative	Fig. 2a, b
HCT-15	CCL-225	ATCC	Correct	Negative	Fig. 2a, b
SW480	CCL-228	ATCC	Correct	Negative	Fig. 2a, b
Capan-1	HTB-79	ATCC	Correct	Negative	Fig. 2a, b
BxPC-3	CRL-1687	ATCC	Correct	Negative	Fig. 2a, b

MDA-MB-231	HTB-26	ATCC	Correct	Negative	Fig. 2a, b
SCaBER	HTB-3	ATCC	Correct	Negative	Fig. 2a, b
CFPAC-1	CRL-1918	ATCC	Correct	Negative	Fig. 2a, b
J82	HTB-1	ATCC	Correct	Negative	Fig. 2a, b
RT4	HTB-2	ATCC	Correct	Negative	Fig. 2a, b
SU.86.86	CRL-1837	ATCC	Correct	Negative	Fig. 2a, b
LoVo	CCL-229	ATCC	Correct	Negative	Fig. 2a, b
PK59	PK59_PANCREAS	NTCC	Correct	Negative	Fig. 2a, b
22Rv1	CRL-2505	ATCC	Correct	Negative	Fig. 2a, b
MDA-MB-468	HTB-132	ATCC	Correct	Negative	Fig. 2a, b
HT-1197	CRL-1473	ATCC	Correct	Negative	Fig. 2a, b
Caco-2	HTB-37	ATCC	Correct	Negative	Fig. 2a, b
PC-3M-2B4	363012	BNCC	Correct	Negative	Fig. 2a, b
1A6	CVCL_6344	NTCC	Correct	Negative	Fig. 2a, b
SK-BR-3	HTB-30	ATCC	Correct	Negative	Fig. 2a, b
KU-19-19	ACC 395	NTCC	Correct	Negative	Fig. 2a, b
647-V	ACC 414	NTCC	Correct	Negative	Fig. 2a, b
UM-UC-3	CRL-1749	ATCC	Correct	Negative	Fig. 2a, b
5637	HTB-9	ATCC	Correct	Negative	Fig. 2a, b
BT-474	HTB-20	ATCC	Correct	Negative	Fig. 2a, b
Hs 578T	HTB-126	ATCC	Correct	Negative	Fig. 2a-b, 5g
HCC 1806	CRL-2335	ATCC	Correct	Negative	Fig. 2a, b
AsPC-1	CRL-1682	ATCC	Correct	Negative	Fig. 2a, b
HCC1428	CRL-2327	ATCC	Correct	Negative	Fig. 2a, b
SW780	CRL-2169	ATCC	Correct	Negative	Fig. 2a, b

MDA-MB-453	HTB-131	ATCC	Correct	Negative	Fig. 2a, b
MDA-MB-157	HTB-24	ATCC	Correct	Negative	Fig. 2a, b
LNCaP	CRL-1740	ATCC	Correct	Negative	Fig. 2a, b
BFTC-905	ACC 361	NTCC	Correct	Negative	Fig. 2a, b
PNT1A	CVCL_2163	NTCC	Correct	Negative	Fig. 2a, b
VCaP	CRL-2876	ATCC	Correct	Negative	Fig. 2a, b
SW1710	ACC-426	NTCC	Correct	Negative	Fig. 2a, b
CCD-18Co	CRL-1459	ATCC	Correct	Negative	Fig. 2g-j, 6p-q, S2a
HeLa	CCL-2	ATCC	Correct	Negative	Fig. 5f
CT-26	CRL-2638	ATCC	Correct	Negative	Fig. 3i-j, S3d,
HEPA1-6	CRL-1830	ATCC	Correct	Negative	Fig. 3k-l, S3e

ATCC: American Type Culture Collection

NTCC: National Typical Culture Collection (Beijing, China)

BNCC: BeNa Culture Collection (Beijing, China)
