# Supplementary material for: Directed natural evolution generates a next-generation oncolytic virus with a high potency and safety profile
Source: Nat Commun. 2023 Jun 9;14:3410. doi: 10.1038/s41467-023-39156-3 (PMC10256765; doi:10.1038/s41467-023-39156-3)
Supplement: Supplementary file 3 — Reporting Summary [file 41467_2023_39156_MOESM3_ESM.pdf]

nature portfolio

Corresponding author(s): Yuan Lin

Last updated by author(s): Jun 1, 2023

Reporting Summary

Nature Portfolio wishes to improve the reproducibility of the work that we publish. This form provides structure for consistency and transparency in reporting. For further information on Nature Portfolio policies, see our Editorial Policies and the Editorial Policy Checklist.

Statistics

For all statistical analyses, confirm that the following items are present in the figure legend, table legend, main text, or Methods section.

n/a	Confirmed	
☐	☒	The exact sample size (*n*) for each experimental group/condition, given as a discrete number and unit of measurement
☐	☒	A statement on whether measurements were taken from distinct samples or whether the same sample was measured repeatedly
☐	☒	The statistical test(s) used AND whether they are one- or two-sided<br>*Only common tests should be described solely by name; describe more complex techniques in the Methods section.*
☒	☐	A description of all covariates tested
☐	☒	A description of any assumptions or corrections, such as tests of normality and adjustment for multiple comparisons
☐	☒	A full description of the statistical parameters including central tendency (e.g. means) or other basic estimates (e.g. regression coefficient) AND variation (e.g. standard deviation) or associated estimates of uncertainty (e.g. confidence intervals)
☐	☒	For null hypothesis testing, the test statistic (e.g. *F*, *t*, *r*) with confidence intervals, effect sizes, degrees of freedom and *P* value noted<br>*Give P values as exact values whenever suitable.*
☒	☐	For Bayesian analysis, information on the choice of priors and Markov chain Monte Carlo settings
☒	☐	For hierarchical and complex designs, identification of the appropriate level for tests and full reporting of outcomes
☒	☐	Estimates of effect sizes (e.g. Cohen's *d*, Pearson's *r*), indicating how they were calculated

Our web collection on statistics for biologists contains articles on many of the points above.

Software and code

Policy information about availability of computer code

Data collection	No customized software was used; NIS-Elements viewer 4.20 was used to collect microscope data; Cell Viability data were collected with Gen 5; CytExpert 2.4 was used to collect flow cytometric data; Applied Biosystems 7500 Step OnePlus real-time PCR system and associated software were used to collect real-time PCR data; Western blot data were collected by Bio-Rad Image lab 5.2.1. Additional information about software was described in the manuscript or available upon request.
Data analysis	No customized software was used for data analysis. Statistical analyses were performed using Graphpad Prism 8 or SPSS 18.0; The microscope images and immumohistochemical staining were analyzed using Image J 1.46r; The sequence analysis were performed with Lasergene software 7.1.0; The Venn diagram was drawn using jveen (http://jvenn.toulouse.inra.fr/app/example.html); The enrichment analysis were performed using metascape (http://metascape.org/gp/index.html#/main/step1).

For manuscripts utilizing custom algorithms or software that are central to the research but not yet described in published literature, software must be made available to editors and reviewers. We strongly encourage code deposition in a community repository (e.g. GitHub). See the Nature Portfolio guidelines for submitting code & software for further information.

Data

Policy information about availability of data

All manuscripts must include a data availability statement. This statement should provide the following information, where applicable:

- Accession codes, unique identifiers, or web links for publicly available datasets
- A description of any restrictions on data availability
- For clinical datasets or third party data, please ensure that the statement adheres to our policy

All data relevant to the study are included in the article or uploaded as supplementary information. The virus genome data are available from the Nucleotide database of National Center for Biotechnology Information under accession number OP683724, OP683725, OP683726 and OP683727. Data can be accessed by these URL:https://www.ncbi.nlm.nih.gov/nuccore/OP683724; https://www.ncbi.nlm.nih.gov/nuccore/OP683725; https://www.ncbi.nlm.nih.gov/nuccore/OP683726; https://www.ncbi.nlm.nih.gov/nuccore/OP683727. The raw data of protein interaction are available from PRIDE database (https://www.ebi.ac.uk/pride/archive/projects/PXD037429). The remaining data are available within the Article, Supplementary Information or Source Data file. Source data are provided with this paper.

Human research participants

Policy information about studies involving human research participants and Sex and Gender in Research.

Reporting on sex and gender	Since no significant difference related to sex was found in the M1 antitumor study, we did not conduct gender analysis.
Population characteristics	The tumor samples are obtained from patients who have not received other treatment.
Recruitment	Primary colorectal carcinoma tissues derived from tumors surgically resected from patients, providing by the Sixth Affiliated Hospital, Sun Yat-sen University. Patients who had received or were receiving other treatments were excluded.
Ethics oversight	All samples were collected with the patients' written informed consent and approved by the ethical review board of the Six Affiliated Hospital of Sun Yat-sen University (no. L2019ZSLYEC-144).

Note that full information on the approval of the study protocol must also be provided in the manuscript.

Field-specific reporting

Please select the one below that is the best fit for your research. If you are not sure, read the appropriate sections before making your selection.

☒ Life sciences ☐ Behavioural & social sciences ☐ Ecological, evolutionary & environmental sciences

For a reference copy of the document with all sections, see nature.com/documents/nr-reporting-summary-flat.pdf

Life sciences study design

All studies must disclose on these points even when the disclosure is negative.

Sample size	For in vitro studies, the experiments were repeated at least 3 times .It is commonly used as the sample size for in vitro studies. For the in vivo experiments,the sample size is determined by the minimum principle of ethical requirements and the tumorigenic capacity of the cancer cells.
Data exclusions	No data were excluded from the data analysis
Replication	All experiments were replicated independently at least typically with technical replicates. Details are indicated in figure legends.
Randomization	For in vivo experiments , randomizing were performed such that the average tumor sizes were similar across treatment groups. Except for experiments involving mice, the samples were randomly divided into experimental and control groups.
Blinding	In in vitro experiments utilizing instrumental readouts such as MTT assay, flow cytometry, Sanger sequencing, and western blot, blinding was not implemented due to the inherent objectivity of the detection instruments. However, in cases of potential bias, such as microscopy analysis or in vivo experiments, data collectors were blinded to the experimental groups.

Reporting for specific materials, systems and methods

We require information from authors about some types of materials, experimental systems and methods used in many studies. Here, indicate whether each material, system or method listed is relevant to your study. If you are not sure if a list item applies to your research, read the appropriate section before selecting a response.

Materials & experimental systems

n/a	Involved in the study
[ ]	[x] Antibodies
[ ]	[x] Eukaryotic cell lines
[x]	[ ] Palaeontology and archaeology
[ ]	[x] Animals and other organisms
[x]	[ ] Clinical data
[x]	[ ] Dual use research of concern

Methods

n/a	Involved in the study
[x]	[ ] ChIP-seq
[ ]	[x] Flow cytometry
[x]	[ ] MRI-based neuroimaging

Antibodies

Antibodies used

anti-Ki-67: Ki-67 (8D5) Mouse mAb (#9449s, Cell Signaling Technology, 1:800), anti-cleaved caspase-3: Cleaved Caspase-3 (Asp175) (5A1E) Rabbit mAb (#9664s, Cell Signaling Technology, 1:2000), anti-GFP : GFP (D5.1) Rabbit mAb (#2956s, Cell Signaling Technology, 1:400), anti-FLAG antibody : Monoclonal antibody -FLAG M2 mouse antibody(#F3165, Sigma); normal mouse IgG : Mouse (G3A1) mAb IgG1 Isotype Control (#5415, Cell Signaling Technology), anti-STAT1: Stat1 (D1K9Y) Rabbit mAb (#14994, Cell Signaling Technology, 1:1000), anti-phospho-STAT1 : Phospho-Stat1 (Tyr701) (58D6) Rabbit mAb (#9167, Cell Signaling Technology,1:1000), anti-PKR : PKR (D7F7) Rabbit mAb (#12297, Cell Signaling Technology, 1:1000), anti-phospho-PKR: Rabbit monoclonal [E120] to PKR (phospho T446) (#ab32036, Abcam, 1:1000), anti-GAPDH : GAPDH (D16H11) XP® Rabbit mAb (#5174, Cell Signaling Technology, 1:1000), anti-E1 and anti-nsP3: mouse mAb (customized by Beijing Protein Innovation,1:1000)

Validation

Ki-67 (8D5) Mouse mAb (#9449s, Cell Signaling Technology) cited in 520 publications; produced by immunizing animals with a synthetic peptide corresponding to residues near the amino terminus of human Ki-67 protein; recommended for detection of Ki-67 of human by Immunohistochemistry (IHC), immunofluorescence (IF) and flow cytometry (F).
Cleaved Caspase-3(Asp 175)(5A1E) Rabbit mAb (#9664s, Cell Signaling Technology) cited in 7194 publications; produced by immunizing animals with a synthetic peptide corresponding to residues of the amino terminal surrounding human caspase-3 Asp175; recommended for detection of Cleaved Caspase-3 of mouse, rat, human and monkey by western blot (WB), immunoprecipitation (IP), IHC, IFand F.
GFP (D5.1) Rabbit mAb (#2956s, Cell Signaling Technology) cited in 653 publications; produced by immunizing animals with a synthetic peptide corresponding to residues of the GFP amino terminal ; recommended for detection of GFP of all species expected by WB and IHC.
Monoclonal antibody -FLAG M2 mouse antibody(#F3165, Sigma) will recognize the FLAG sequence at the N-terminus, Met-N-terminus, C-terminus, or at an internal site of FLAG fusion proteins. It is Purified IgG1 subclass. Monoclonal ANTI-FLAG M2 is useful for identification and capture of FLAG fusion proteins by common immunological procedures such as WB and IP of all species expected.
Mouse (G3A1) mAb IgG1 Isotype Control (#5415, Cell Signaling Technology) cited in 317 publications. Mouse (G3A1) mAb IgG1 Isotype Control does not target any known antigen. It acts as a homologous control against murine IgG1 monoclonal antibody, and used to evaluate non-specific binding of target primary antibodies due to Fc receptor binding or other protein-protein interactions. The type and concentration of immunoglobulin used for homologous control antibodies should be the same as for detection antibodies.
Stat1 (D1K9Y) Rabbit mAb (#14994, Cell Signaling Technology) cited in 346 publications; produced by immunizing animals with a synthetic peptide corresponding to residues surrounding Pro688 in human Stat1 protein; recommended for detection of STAT1 of mouse, rat, human and monkey by WB, IP, IHC, IF, F and Chromatin immunoprecipitation (ChIP).
Phospho-Stat1 (Tyr701) (58D6) Rabbit mAb (#9167, Cell Signaling Technology) cited in 875 publications; produced by immunizing animals with a synthetic phosphopeptide corresponding to residues surrounding Tyr701 in human Stat1; recommended for detection of Phospho-STAT1 of mouse and human by WB, IP, IHC, IF, F and Chromatin immunoprecipitation (ChIP).
PKR (D7F7) Rabbit mAb (#12297, Cell Signaling Technology) cited in 61 publications; produced by immunizing animals with a synthetic peptide corresponding to residues surrounding Leu222 in human PKR protein; recommended for detection of PKR of human by WB and IP.
Rabbit monoclonal [E120] to PKR (phospho T446) (#ab32036, Abcam) cited in 107 publications; Produced recombinantly (animal-free) for high batch-to-batch consistency and long term security of supply; recommended for detection of Phospho-PKR of human and pig by WB,IHC and IP.
GAPDH (D16H11) XP Rabbit mAb (#5174, Cell Signaling Technology) cited in 5800 publications; produced by immunizing animals with a synthetic peptide corresponding to residues near the carboxyl terminus of human GAPDH; recommended for detection of GAPDH of mouse, rat, human and monkey by WB, HC and IF.
Anti-E1 and anti-nsP3 antibodys were customized using the whole protein by Beijing Protein Innovation, and were used according to previous experience. There are mouse monoclonal antibodies and well validated by western blots. Our research team has demonstrated that their target bands are clear in several published papers (DOI: 10.1126/scitranslmed.aam7996; DOI: 10.1073/pnas.1408759111; DOI: 10.1038/mt.2015.172; DOI: 10.18632/oncotarget.10305; DOI: 10.1073/pnas.1701002114;DOI: 10.1089/hum.2017.055; DOI: 10.1128/JVI.01331-17; DOI: 10.1038/s41467-018-03913-6; DOI: 10.1038/s41467-018-06771-4).

Eukaryotic cell lines

Policy information about cell lines and Sex and Gender in Research

Cell line source(s)

Sk-HEP-1, HCT-116, ZR-75-1, MCF-7, Capan-2, BT-20, MDA-MB-435S, HPAC, PANC-1, HT-29, MIA PaCa-2, HCC38, HT-1376, SW620, SW 1990, T-47D, DU 145, HCT-15, SW480, Capan-1, BxPC-3, MDA-MB-231, SCaBER, CFPAC-1, J82, RT4, SU.86.86, LoVo, 22Rv1, MDA-MB-468, HT-1197, Caco-2, SK-BR-3, UM-UC-3, 5637, BT-474, Hs 578T, HCC 1806, AsPC-1, HCC1428, SW780, MDA-MB-453, MDA-MB-157, LNCaP, VCaP, CCD-18Co, HeLa, CT-26, HEPA1-6 were initially obtained from the American Type Culture Collection. HCT-8, Huh 7, PC-3M-2B4 were purchased from BeNa Culture Collection. RT112/84, VM-

	CUB-1, PK59, 1A6, KU-19-19, 647-V, BFTC-905, PNT1A, SW1710 were purchased from National Typical Culture Collection. Except for HCT-116, HCT 8, Huh 7, CCD-18Co, SW620, CT-26,HEPA1-6, HeLa and Hs 578T, the remaining cell lines are only mentioned in figure 2A of the manuscript.<br>HeLa-Mxra8 were generated by transducing HeLa cells with the Mxra8-expressing lentiviral vector (Deli Song et al. STTT, 2022).Hs 578T-ΔMxra8 were generated by using CRISPR-Cas9 gene editing technology (Deli Song et al. STTT, 2022).
Authentication	All cell lines were authenticated by STR profiling. Details are shown in supplementary table S8.
Mycoplasma contamination	All cell lines used in experiments were tested negative for mycoplasma contamination. Details are shown in supplementary table S8.
Commonly misidentified lines (See ICLAC register)	No commonly misidentified cell lines were used.

Animals and other research organisms

Policy information about studies involving animals; ARRIVE guidelines recommended for reporting animal research, and Sex and Gender in Research

Laboratory animals	Four- to six-week-old female BALB/c-nude mice (Beijing Vital River Laboratory Animal Technology Co., Ltd.) were housed in a pathogen-free room in the experimental animal center of Sun Yat-sen University. Six- to eight-week-old female BALB/c mice and C57BL/6 mice were housed in pathogen-free room in Lani Scientific (Guang Zhou) Co., Ltd.. Mice were housed at an ambient temperature of 22-24 ℃, humidity-controlled environment at 40%-70% under a 12-hour light/dark cycle with ad libitum access to water and food.<br>Eight captive-bred 4-5-year-old pathogen-free cynomolgus macaques (four males and four females) were reared and handled at JOINN Laboratories (China) Co., Ltd..
Wild animals	No wild animals were used in this study.
Reporting on sex	Female mice in different litters are usually less likely to fight and easier to be raised in groups, which is conducive to the growth of subcutaneous tumors and random grouping. We did not conduct a gender-based analysis in the nonhuman primate study because we included an equal distribution of male and female Macaques and the results of our previous and current study did not show any significant differences related to sex.
Field-collected samples	No field-collected samples were used.
Ethics oversight	The mouse experiments were performed under the protocol approved by the Animal Ethics and Welfare Committee of Sun Yat-sen University (no. 2016-114) and the Laboratory Animals Ethics Committee of Lani Scientific (Guang Zhou) Co., Ltd. (no. G2022024). The non-human primates used in this study were approved by the Institute's Animal Management and Use Committee (IACUC no ACU18-1162), and the experiments were conducted according to the Guide for the Care and Use of Laboratory Animals, 8th Edition.

Note that full information on the approval of the study protocol must also be provided in the manuscript.

Flow Cytometry

Plots

Confirm that:

- [x] The axis labels state the marker and fluorochrome used (e.g. CD4-FITC).
- [x] The axis scales are clearly visible. Include numbers along axes only for bottom left plot of group (a 'group' is an analysis of identical markers).
- [x] All plots are contour plots with outliers or pseudocolor plots.
- [x] A numerical value for number of cells or percentage (with statistics) is provided.

Methodology

Sample preparation	HCT-116, HeLa, Hs 578T and Hs 578T-ΔMxra8 cells were infected with M1viruses for 48h, and the infection rate was determined by detecting the expression of GFP during viral replication.
Instrument	CytoFLEX, Beckman Coulter
Software	CytExpert 2.4
Cell population abundance	The purity of sorted cells was over 99% because there's no sorting or purification,
Gating strategy	To determine GFP expression in tumor cells, live single cells were further gated on FSC-A/SSC-A, FSC-A/FSC-H, and then the expression level of GFP was detected using FITC channels.

- [x] Tick this box to confirm that a figure exemplifying the gating strategy is provided in the Supplementary Information.
